# Stereoselective Double Reduction of 3-Methyl-2-cyclohexenone, by Use of Palladium and Platinum Nanoparticles, in Tandem with Alcohol Dehydrogenase

**DOI:** 10.3390/nano8100853

**Published:** 2018-10-19

**Authors:** Francesca Coccia, Lucia Tonucci, Piero Del Boccio, Stefano Caporali, Frank Hollmann, Nicola d’Alessandro

**Affiliations:** 1 Department of Engineering and Geology (INGEO), G. d’Annunzio University of Chieti-Pescara, Viale Pindaro 42, I-66100 Chieti Scalo, Italy; frasmile.f@libero.it; 2 Department of Biotechnology, Delft University of Technology, van der Maasweg 9, 2629HZ Delft, The Netherlands; f.hollmann@tudelft.nl; 3 Department of Philosophical, Educational and Economic Sciences, G. d’Annunzio University of Chieti-Pescara, Via dei Vestini 31, I-66100 Chieti Scalo, Italy; lucia.tonucci@unich.it; 4 Department of Pharmacy, G. d’Annunzio University of Chieti-Pescara, Via dei Vestini 31, I-66100 Chieti Scalo, Italy; piero.delboccio@unich.it; 5 Department of Chemistry, University of Firenze, Via della Lastruccia 3-13, I-50019 Sesto Fiorentino, Italy; stefano.caporali@unifi.it

**Keywords:** nanoparticles, biocatalysis, palladium, platinum, chemoenzymatic catalysis, alcohol dehydrogenase, tandem reaction, nanocatalysis

## Abstract

The combination of metal nanoparticles (Pd or Pt NPs) with NAD-dependent thermostable alcohol dehydrogenase (TADH) resulted in the one-flask catalytic double reduction of 3-methyl-2-cyclohexenone to 3-(1S,3S)-methylcyclohexanol. In this article, some assumptions about the interactions between a chemocatalyst and a biocatalyst have been proposed. It was demonstrated that the size of the NPs was the critical parameter for the mutual inhibition: the bigger the NPs, the more harmful for the enzyme they were, even if the NPs themselves were only moderately inactivated. Conversely, the smaller the NPs, the more minimal the TADH denaturation, although they were dramatically inhibited. Resuming, the chemocatalysts were very sensitive to deactivation, which was not related to the amount of enzyme used, while the inhibition of the biocatalyst can be strongly reduced by minimizing the NPs/TADH ratio used to catalyze the reaction. Among some methods to avoid direct binding of NPs with TADH, we found that using large Pd NPs and protecting their surfaces with a silica shell, the overall yield of 3-(1S,3S)-methylcyclohexanol was maximized (36%).

## 1. Introduction

One-flask cascade reactions constitute a fascinating branch of organic chemistry, closely mimicking natural biosynthetic pathways. The advantage of one-flask cascade reactions over multistep processes has shown that transformations can be performed without any separation and purification steps of the intermediates, thus minimizing waste, equipment, energy, and costs [1,2,3,4,5].

In a recent review, Anastas and co-workers put the one-pot synthesis as one “leaf” of their “Green ChemisTREE” whose branches represent the principles of green chemistry and the leaves are the techniques that can be used to follow and to realize them [6].

By using a combination of chemo- and biocatalytic routes, this provides a particularly attractive method for organic synthesis [7,8,9]. Chemical catalysis using transition metals has been shown to be highly versatile, efficient, and manageable, but very often lacks chemo and enantioselectivity, whereas enzymatic systems are highly selective, but often less versatile, and more selective for a narrower range of substrates, while also much less stable than transition metal catalyst to harsher conditions [10,11,12,13]. Less effort has been devoted to the development of a toolkit for chemical and biological catalysts working in tandem, since the general reaction conditions were often found to be contrary to normal conditions or were detrimental to enzyme stability [14,15]. This topic has great interest, since bioconjugated nanomaterials are finding increasing applications in biotechnology [16,17,18], as biosensors [19,20,21] and for drug delivery [22,23].

Thanks to the recent success in mimicking nature, by using mild conditions, e.g., green solvents [24,25], chemical reactions conducted in water media [26,27], etc., it has become possible to effectively combine chemo and biocatalytic routes, for example the pioneering study of reduction of glucose to mannitol, performed by a combination of the isomerase enzyme and a copper catalyst [28], and, more recently, a combination of alcohol dehydrogenase (ADH) with lignin-stabilized Pt or Pd nanoparticles (NPs), which was able to catalyze the synthesis of enantiomerically pure 1,2-amino alcohols [29].

**Scheme 1:**
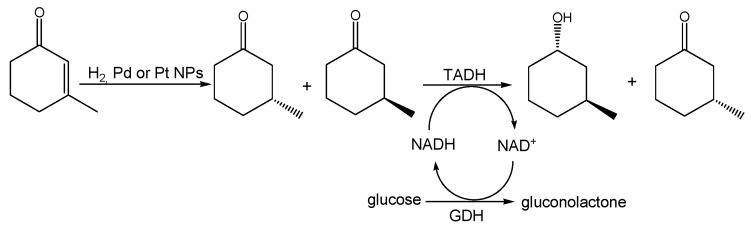
Reduction of the C=C double bond with Pd or Pt NPs in tandem with the asymmetric reduction of the keto group by TADH. The reaction was performed under 10 bar H_2_ at 30 °C in phosphate buffered solution, using glucose dehydrogenase (GDH) and glucose as the nicotinamide cofactor (NADH) regeneration system.

In the present study we chose Pd and Pt NPs as the metal precatalyst and a NAD^+^-dependent thermostable alcohol dehydrogenase, extracted from *Thermus* sp. ATN1 (TADH), as the biocatalyst to perform a significant example of a stereoselective one-flask tandem reduction of 3-methyl-2-cyclohexenone to 3-(1S,3S)-methylcyclohexanol (Scheme 1). The Pd and Pt NPs have been shown to be excellent catalysts, due to their high surface-area-to-volume ratio, easy preparation, and good “solubility” in water, where they can work without significant pH or temperature limitations and have been used in oxidation, reduction, and cross-coupling reactions [30,31,32]. TADH has shown a broad substrate spectrum, which includes aldehydes, aliphatic ketones, cyclic ketones, and double-ring systems [33,34].

In this paper, we have focused on the possible methods to minimize the strong mutual inhibition of the chemo and biocatalyst; the effects of NPs on the structures of proteins, and vice versa, have not been fully understood, despite the many studies dealing with the structure change, and residual activity of biological molecules adsorbed on the surface of nanoscale materials [35,36].

## 2. Results and Discussion

The overall chemoenzymatic cascade reaction we speculated on is shown in Scheme 1. We envisioned a chemical hydrogenation of conjugated C=C double bond in 3-methyl-2-cyclohexenone catalyzed by lignin-stabilized Pd or Pt NPs, yielding 3-methylcyclohexanone, followed by an enzymatic kinetic resolution of the racemic intermediate to be enantioselective, with respect to the absolute configuration of the methyl substituent, and enantiospecific, with respect to the absolute configuration of the alcohol formed in the reduction. It is worth noting that, despite the many enoate reductases known today, which in principle could also mediate the first step, there has not yet been an efficient enoate reductase known to recognize β-substituted enones.

In the initial experiments, we investigated the chemical hydrogenation step, i.e., the reduction of 3-methyl-2-cyclohexenone to (racemic) 3-methylcyclohexanone using only Pt or Pd NPs catalysts. As shown in Table 1, the Pt catalyst excelled in terms of reaction rate and conversion of the starting material, but also exhibited selectivity issues for the over reduction product 3-methylcyclohexanol, especially at elevated hydrogen pressures. Moreover, the Pd catalyst was somewhat less active, but exhibited high chemoselectivity (mostly at high H_2_ pressure) for the reduction of the C=C-bond.

We next performed the cascade reaction shown in Scheme 1 but in a two-step mode: after performing the chemical hydrogenations, the reaction mixtures were supplemented with the alcohol dehydrogenase from *Thermus* sp. ATN1 (TADH), catalytic amounts of the 1,4-dihydronicotinamide cofactor (1,4-NADH) and a suitable in situ regeneration system, using glucose dehydrogenase (GDH) and glucose. When Pd NPs were employed, the overall reaction gave 95% conversion and 47% yield of 3-(1S,3S)-methylcyclohexanol and the diastereomeric excess, measured by gas chromatography (GC), was 95%, in agreement with previous literature data [37]. Moreover, with Pt NPs, conversions were almost quantitative, whereas the yield of 1S,3S alcohol was significantly lower, in agreement with the selectivity value obtained for the Pt NPs catalyzed hydrogenation (see above); the final reaction mixture provided (R)-3-methylcyclohexanone (46%) and all the possible stereoisomers, i.e., (1S,3S)-(42%), (1R,3R)-(4%), (1R,3S)-(4%) and 3-(1S,3R)-methylcyclohexanol (4%).

The reaction was finally carried out in the one-flask mode, with both catalysts (metal NPs and TADH) present. With the Pd NPs/TADH combination, a 42% conversion was observed, with a very low yield of (1S,3S)-3-methyl-cyclohexanol (14%), whereas with Pt NPs, no reaction was observed, therefore, clearly indicating mutual inhibition of the catalysts.

**Figure 1:**
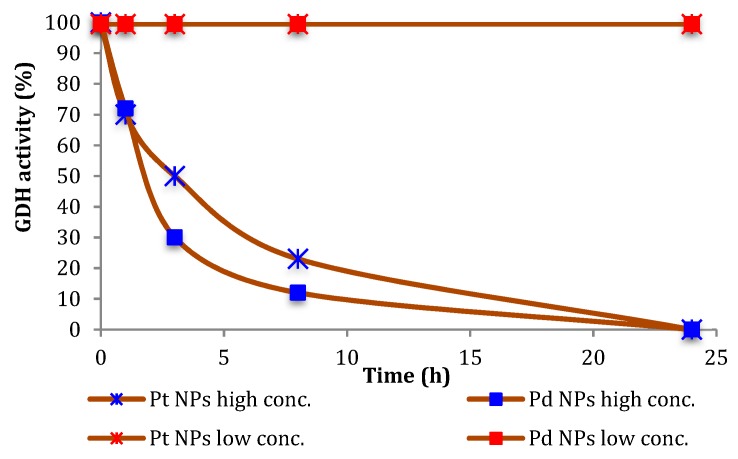
GDH activity over time in the presence of Pt and Pd NPs, at different NPs concentrations. (NPs low conc.: 10 μL of 5.64 × 10^−3^ M Pd NPs or 5 μL of 9.66 × 10^−3^ M Pt NPs, with 4 mg of GDH similar to the same ratio used in the reaction carried out in autoclave; NPs high conc.: 100 μL of 5.64 × 10^−3^ M Pd NPs or 50 μL of 9.66 × 10^−3^ M Pt NPs, with 4 mg of GDH) (see experimental).

Therefore, we investigated the effect of the NPs on the stability of the biocatalysts. As shown in Figure 1, the inactivation of GDH under the conditions for the above-mentioned experiments was not very significant, and a time-dependent inhibition of GDH was observed only in the presence of 10-fold higher NPs concentrations.

**Figure 2:**
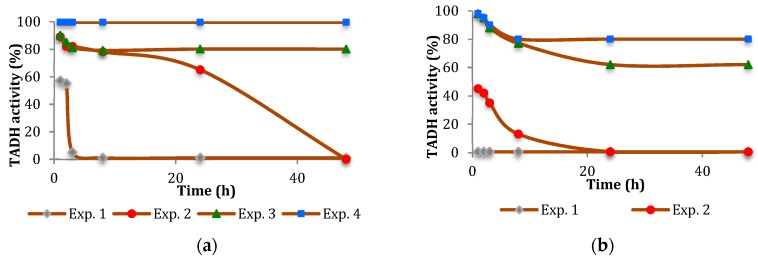
TADH activity (%) versus incubation time (hours) in samples made by Pt NPs incubated with different TADH volumes; NPs were of two different dimensions: (**a**) 8 nm; (**b**) 19 nm. The experimental conditions were: Exp. 1, 10 μL of Pt NPs solution and 50 μL of TADH; Exp. 2, 10 μL of Pt NPs and 100 μL of TADH; Exp. 3, 10 μL of Pt NPs solution and 800 μL of TADH; Exp. 4, 10 μL of Pt NPs and 3 mL of TADH (see experimental).

**Figure 3:**
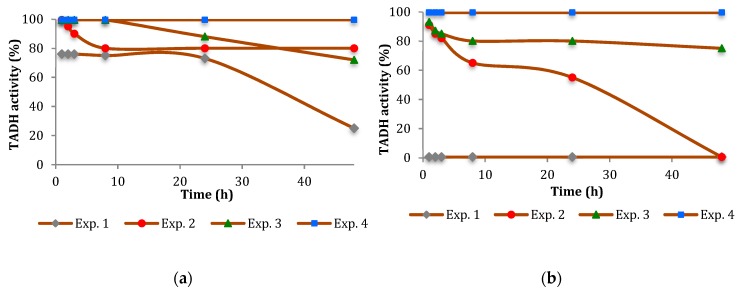
TADH activity (%) versus incubation time (hours) in samples made by Pd NPs incubated with different TADH volumes; NPs were of two different dimensions: (**a**) 8 nm; (**b**) 14 nm. The experimental conditions were: Exp. 1, 20 μL of Pd NPs solution and 50 μL of TADH; Exp. 2, 20 μL of Pd NPs and 100 μL of TADH; Exp. 3, 20 μL of Pd NPs solution and 800 μL of TADH; Exp. 4, 20 μL of Pd NPs and 3 mL of TADH (see experimental).

The inhibition of TADH was more pronounced (Figure 2 and Figure 3). In general, the inhibitory effect of the NPs tested was time-dependent, indicating a relatively slow inhibition mechanism, with Pt more inhibitory than Pd. Furthermore, the extent of inhibition appeared to depend on the NPs/TADH ratio.

Several alternative mechanisms have been reported trying to explain the mutual inhibition between metal catalysts and enzymes [38]. The adsorption of organics onto solid surfaces primarily depends on the geometry and the physicochemical characteristics of the solid surface itself [39], in particular size and shape [40]. The Pd and Pt NPs can be obtained, by changing the reaction time of the synthetic procedure, with different particle size. While the shape of the Pd NPs employed were always spherical, the Pt NPs were in irregular shape [30].

Comparing the activity on the TADH enzyme by the presence of Pt and Pd NPs having comparable dimensions (i.e., ≅ 8 nm, as evidenced by Dynamic light scattering (DLS) measurements), we found that Pt NPs always acted as a stronger inhibitor than the spherical Pd NPs (Figure 2a and Figure 3a). Comparing the NPs of the same metal, but with different dimensions (i.e., 8 nm and 19 nm for Pt NPs; 8 nm and 14 nm for Pd NPs), we noted that, in both cases, the denaturing effect on the protein became stronger by increasing the dimension of the NPs (Figure 2 and Figure 3).

**Figure 4:**
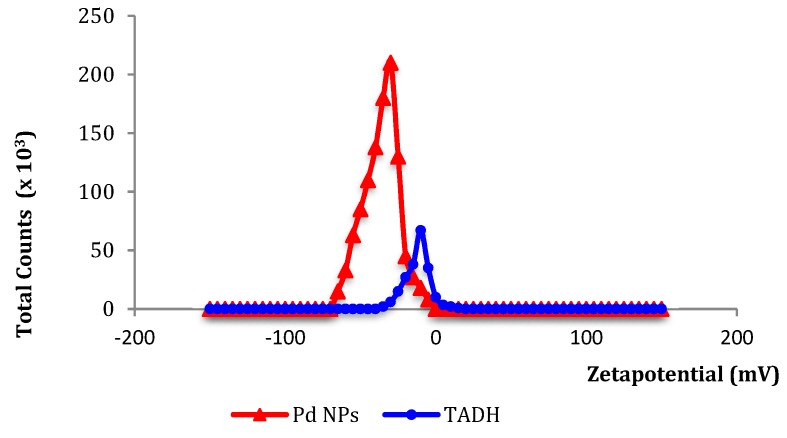
Distribution of the net charge of TADH (−10 mV) and Pd NPs (−28 mV).

Specific and non-specific interactions, such as electrostatic, hydrogen bonding and hydrophobic forces, could critically affect the structure, activity, and stability of the proteins, once they are adsorbed onto NPs surfaces or vice versa, NPs adsorbed onto protein surfaces [35]. The small net negative charges (from zeta potential data: Figure 4) measured for both Pd NPs and TADH strongly suggest that electrostatic forces are less important than hydrophobic interactions for the mutual inhibition.

For a better insight into the inhibition of NPs in the presence of TADH, the reactions were carried out at various TADH/NPs ratios, and also by using NPs previously incubated with TADH; it was concluded that the inhibition of NPs did not depend on either the enzyme/NPs ratio, nor on the incubation time; the yields of 3-methylcyclohexanone in the one-flask reaction does not significantly change with the amount of enzyme used, and the same results were observed when different incubation time were tested. Instead, the larger and quasi spherical Pd NPs resulted in less significant deactivation, and thus, we will only take into consideration the Pd NPs.

**Figure 5:**
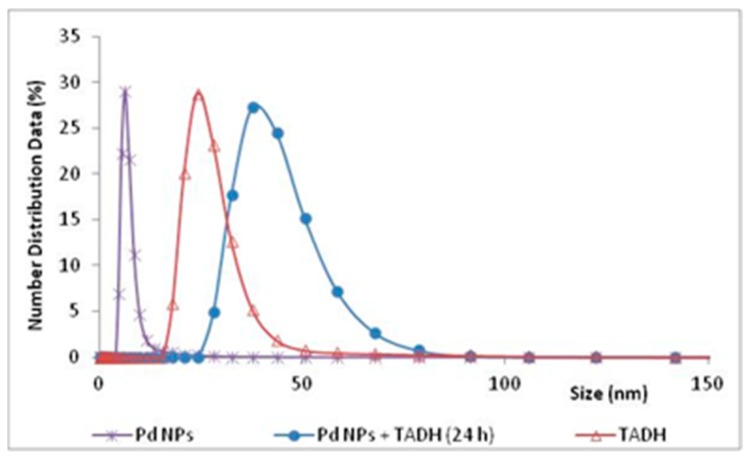
Dynamic light scattering (DLS) spectra of diluted solution of Pd NPs (8 nm), phosphate-buffer-diluted solution TADH (24 nm) and diluted solution Pd NPs after a 24 h incubation period with TADH (38 nm).

The experimental evidence for an effective adsorption interaction, between the metal NPs and the protein, was clearly given by DLS measurements, which signaled a significant increase in the size of the NPs, after a 24 h incubation with TADH (Figure 5).

**Figure 6:**
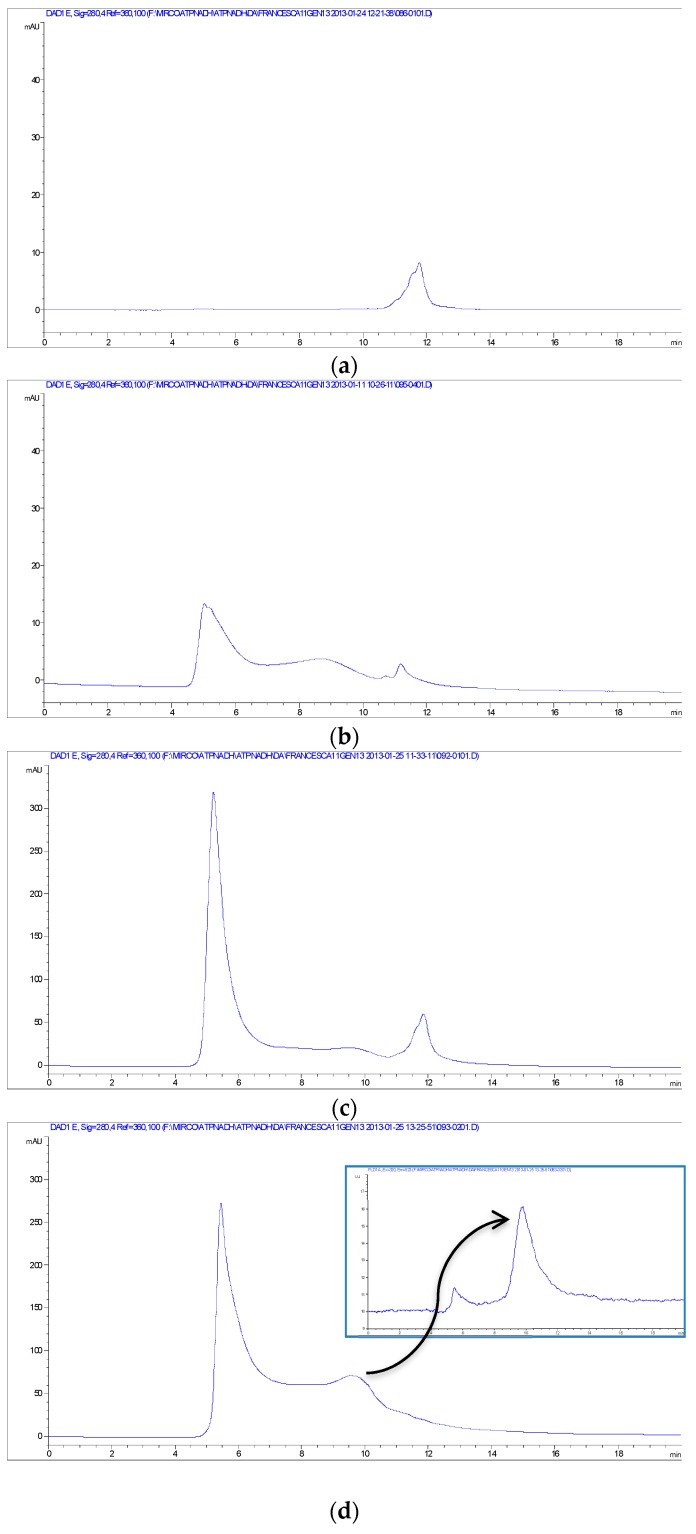
From the top to the bottom: Size-exclusion chromatogram of (**a**) TADH solution; (**b**) TADH solution incubated with NPs (100 μL TADH solution: 5 μL Pd NPs solution); (**c**) TADH solution incubated with NPs (100 μL TADH solution: 15 μL Pd NPs solution); (**d**) TADH solution incubated with NPs (100 μL TADH solution: 25 μL Pd NPs solution) with its fluorescence data (inset).

Other evidence for the NPs-TADH interactions was obtained from size-exclusion chromatography-HPLC analyses (SEC), using both UV-Vis and fluorescence detection. The chromatogram of the solution of TADH (~40 kDa [33]) exhibited a single peak at 11.7 min (Figure 6a), which was also present when the fluorescence was set to reveal tryptophane; therefore, attributable to a protein structure. Instead, the SEC chromatogram of the solution of TADH after 24 h incubation with Pd NPs (100 μL of TADH 250 μg/mL and 5 μL of Pd NPs) exhibited a weak signal at 11.7 min, indicative of the presence of minor amounts of free TADH, and two intense peaks at 5 min (solvent front) and in the 7–10 min range (Figure 6b), indicating that many different shifts of the TADH mass have occurred. By increasing the amount 3X of the Pd NPs, the chromatogram of the incubated TADH showed, besides a very weak peak at 11.7 min, an intense one at the solvent front (Figure 6c), that indicated that the larger amounts of added NPs triggered a significant growth in size of the nano-bioconiugates, which were almost entirely eluted at the beginning of the chromatographic course; the column could not separate very large masses, of over 100 KDa. When TADH was incubated with even larger amounts of NPs (up to 5X Pd NPs), the complete disappearance of the free TADH peak at 11.7 min occurred, with the concomitant appearance of intense signals, both at the solvent front and between 7.5 and 10.5 min (Figure 6d); this last peak cannot be attributed to NPs, as the fluorescence signal helped to identify it as a protein (see the insert on Figure 6d), and it was concluded that complete aggregation of the protein with the NPs occurred, with the formation of a variety of nano-bioconjugates with different sizes. The focus was to determine the strength of NPs-protein interactions, for fractions responsible for the intense peak showed in Figure 6d, and falling between 5 and 7.5 min. The recovered fractions were re-injected in the SEC apparatus; the same peaks were indeed detected in the chromatogram, with the same area, strongly suggesting that, once formed, the nanobioconjugate did not release the free enzyme. The recovered sample was tested for the reduction of 3-methylcyclohexanone to (1S,3S)-3-methylcyclohexanol, and resulted in complete inactivity, thus indicating that the carbonyl reduction was effected only by free TADH, and not from Pd NPs-TADH adducts.

In our optimized experimental conditions for the quantitative C=C double bond catalytic reduction, the amount of metal NPs were always noticeably lower than the TADH enzyme; under these conditions, TADH never completely aggregated to the NPs, and thus, explaining why there was a TADH activity of only a 20% inhibition.

Centrifuge filters, with 100 kDa cut-off, were used to isolate the formed nano-biococonjugate. The filter permeability was tested for TADH and for Pd NPs. TADH passed through the filter, as its weight is ~ 40 kDa, while Pd NPs were clearly retained; the solution became colorless, and the filter turned dark-brown, thus indicative that a mechanical recover of the solid fraction from the filter was feasible. It was also likely that by filtering the TADH solution incubated with NPs, the nanobioconjugate adduct could be collected on the filter, whereas the free TADH was removed with the filtrate. Therefore, using the concentration of Pd NPs, 3X/5X times higher than the one usually employed in the one-flask reaction, we expected to achieve the complete disappearance of free TADH, and its complete aggregation with NPs. Indeed, the ninhydrine test was negative for the solution, as was the Bradford test, performed on the wash solution of the residual on the filter. In contrast, when we carried out the Bradford test on an aliquot of solid residue, the test was positive, thus confirming that the protein remained quantitatively on the filter, and adsorbed to the NPs.

In this manner, it was possible to obtain a solid sample for the X-ray photoelectron spectroscopy analysis (XPS), which is a very useful tool to investigate the atomic composition and chemical environment of the outermost few nanometer layers of a surface (~10 nm), and to accurately determine the surface area [41,42].

**Figure 7:**
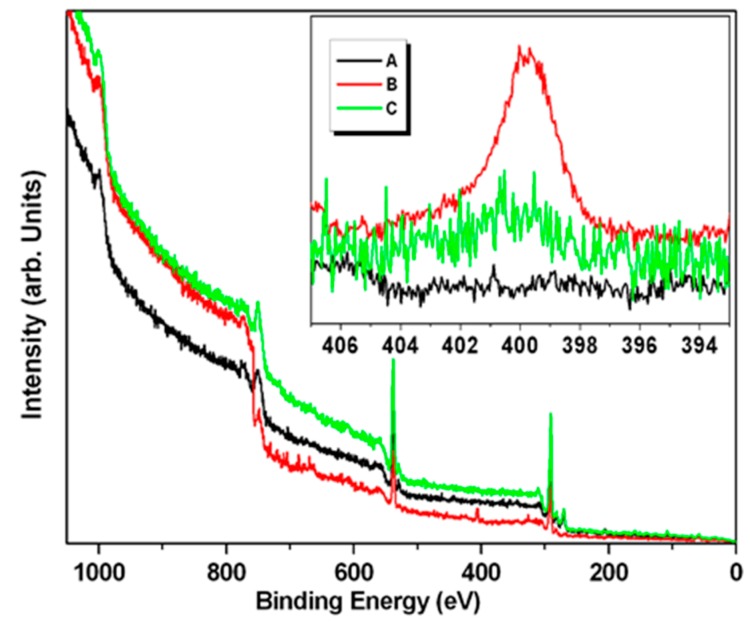
XPS spectra of Pd NPs powder alone (black, A) and Pd NPs incubated with TADH (100 μL) respectively with 15 μL of Pd NPs solution, 3X the standard one-flask conditions (red, B) and 25 μL of Pd NPs solution, 5X the standard one-flask conditions (green, C). Insets repost the details of the XPS spectra (characteristic region of nitrogen 1s core transition).

The wide scan XPS spectrum of the Pd NPs powder was dominated by the peak attributable to the carbon 1s signal at 285 eV, and that of oxygen at 530 eV, where both arise from the lignin structure (Figure 7-black); while the spectrum of the sample of NPs incubated with TADH (3X the amount used in the standard one-flask reaction) showed a new signal attributable to nitrogen (1s core transition at 400 eV), a clear indication for the presence of the protein (Figure 7-red), which disappeared for a sample more rich in NPs (up to 5X the amount used in the standard one-flask reaction) (Figure 7-green). The region of spectrum characteristic of the nitrogen 1s core transition (392–408 eV) is displayed in Figure 7-inset. As expected, the peak attributable to nitrogen was not detected in the Pd NPs powder sample. Thus, nitrogen was detected in the spectra collected on samples incubated with TADH (Figure 7-inset red and green), even if in the experiments conducted on the more concentrated Pd NPs sample (green line), the intensity was very weak. The presence of nitrogen in these samples was confirmed by the positive ninhydrin test for the same sample containing TADH, and the negative one for the NPs sample. Therefore, it was possible to conclude that when NPs is present in large amounts, more NPs are adsorbed onto the protein, resulting in full coverage, and shielding of TADH. However, it should be remebered that the XPS is a surface technique, and does not reveal the composition under the lignin-metal shell.

The consequence of the formation of stable nano-bioconjugates is that a partial/total deactivation of both the metal NPs and the TADH occurs. It was very likely that TADH could acts as biocatalyst only when completely free from the interaction with the metal NPs, the aggregated fraction being completely denaturated by the metal linkage. As for the inhibitory role of the enzyme on the catalytic behaviour of NPs, one can only speculate that when the metal NPs become bound to the protein, their exposure to the substrate must definitely decrease.

**Figure 8:**
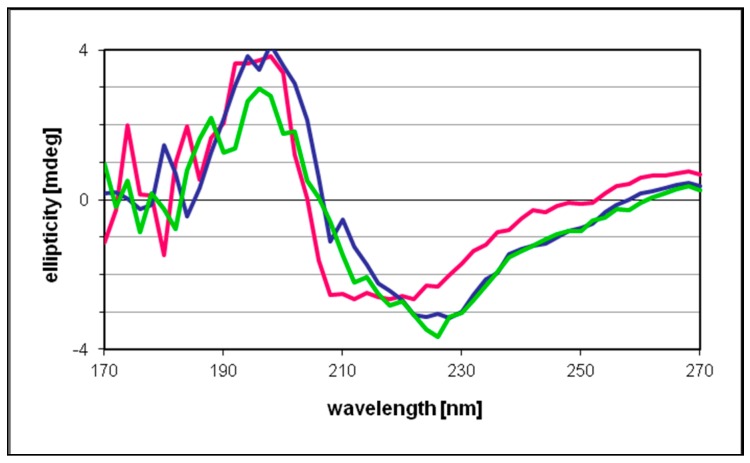
Circular dichroism spectrum recorded for a TADH sample before (pink line) and after 24 h at 30 °C (dark blue line), and after 24 h at 30° C in the presence of Pd NPs (green line).

The circular dichroism data recorded for free and NPs-bonded protein were essentially identical, indicating only marginal changes in the protein secondary structure upon incubation with the metal catalyst (Figure 8). These results also suggested that under the present reaction conditions the proportion of the enzyme involved in the binding with the NPs was rather low, therefore scarcely affecting the “average” secondary structure of TADH. It would have been interesting to record a spectrum at higher NPs concentrations, as done in SEC and XPS experiments, but beyond 20 μL/mL Pd NPs concentration, the absorbances were too high and data points below 210–220 nm were unreliable.

An obvious solution to mitigate the mutual inactivation of NPs and biocatalysts was their physical separation. Several different approaches were evaluated: (i) immobilization of TADH in sol-gel, with sols from traditional silicates or diglycerylsilane (DGS) [43]; (ii) immobilization of Pd NPs in sol-gel (DGS); (iii) encapsulation of Pd NPs [44] in DGS [45,46], or traditional silica coatings [47].

Thus, by using the traditional silicate sol-gel of TADH, the efficiency of the Pd NPs increased notably, leading to a 95% yield of racemic 3-methylcyclohexanone, but, unfortunately, the sol-gel immobilization affected the activity of the enzyme (24% less active), possibly because the sol-gel hardness did not allow the natural conformational changes of the protein [48]. Alternatively, severe diffusion limitations of the nicotinamide cofactor, which has to diffuse between GDH and TADH, may account for the poor efficiency of the enzymatic reduction. Sol-gels with different times of aging were tested; however, those used immediately became brown at the end of the reaction, indicating that NPs, present in the solution outside the sol-gel, went inside. Instead, the sol-gel that had been kept for one day in the fridge, appeared brown at the end of the reaction, only on its surface, which means that it was harder to be penetrated by Pd NPs. Therefore, a less harsh sol-gel from tetraethyl orthosilicate (TEOS) with bigger pores was prepared by adding Mg^2+^ salt; in this situation, even after the curing time of one day in the fridge, the sol-gel became the same color of the NPs at the end of the reaction, which signifies that this kind of sol-gel was permeable to the Pd NPs, and probably to the enzyme. A series of different sol preparations, using different silica precursors (TEOS; tetramethyl ortosilicate—TMOS; methyl trimethoxysilane—MTMS; 3-aminopropyltrimethoxysilane—APTES) and different dilution ratios (sol/sample solution), were tested, but no significant improvements were observed, at least in terms of yields of the enzymatic reactions.

Another silica precursor tested was DGS, a sugar functionalised by silicate, particularly advantageous, since the percentage shrinkage of its sol-gel is low and the condensation of the gel occurs at neutral pH. Extreme conditions are not necessary to synthesize DGS, and a polyol such as glycerol, can stabilize the protein [45,46]. However, by entrapping the enzyme in a DGS sol-gel, the results were obtained were no better, since again, the loss of enzymatic activity was ~24%, which must be added to a 10% loss of NPs activity.

As the most sensitive catalyst to the inhibition was the chemocatalyst, we tested its immobilization in the sol-gel, thus limiting any direct interaction with the enzyme. The Pd NPs was entrapped in a DGS sol-gel, which has smaller pores (1.56 nm) [46] than those of the traditional sol-gel: the Pd NPs worked very well, although a bit slower, but the decrease of the enzymatic efficiency was still around 24%, possibly due to the interactions of TADH with the large and flat surface of the sol-gel, which the enzyme comes in contact. This finding could be explain by the concepts formulated by Dordick and his co-workers, where the enzymatic inactivation depends on the dimensions of the NPs and the surface curvature; i. e., the larger the NPs and the less curvature they have, the stronger the denaturation of the native enzyme structure [49].

**Figure 9:**
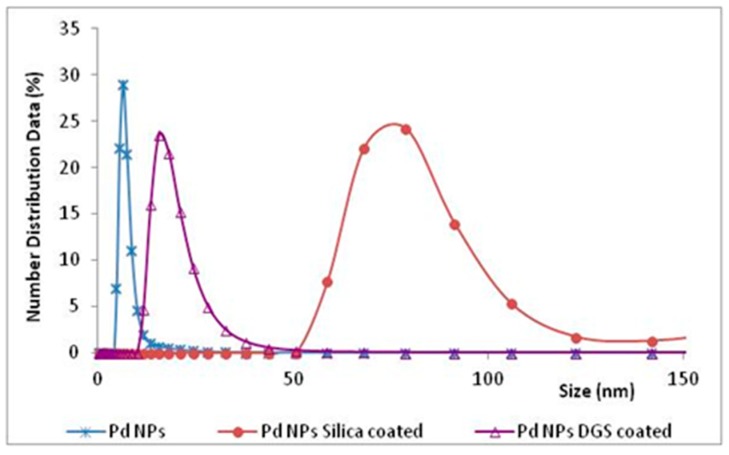
DLS spectra of Pd NPs solution (8 nm); DGS-coated Pd NPs (16 nm); silica-coated Pd NPs (80 nm).

Another possibility to protect NPs was that to build a shell around them, by using silica precursors for the coating process, in order to obtain silica hollow sphere containing several mono-dispersed metal NPs inside [50]. DGS was again used to prepare a very thin silica shell, that was not dependent upon the DGS concentration or the incubation time [45,46]. This coating was assisted by the lignin of the NPs, that acted as a template on which the DGS was hydrolysed, and favored to form networks stabilized by hydrogen bonding interactions between silanols and methoxy groups of lignin. The size of the “naked” NPs was 8 nm, while the final diameter of the same NPs covered by the DGS layer was 16 nm. However, the covering of NPs with a thin layer of DGS was not critical, when comparing the results with those obtained by the “naked” Pd NPs, possibly due to the shells being too thin to change the chemical and physical characteristics of NPs, and avoid their ‘hiding’ on protein surface. Traditional silica precursors, such as TEOS and APTES, added immediately after the NPs formation, gave silica shell NPs with an average diameter of 80 nm (DLS measurements, Figure 9). In this manner, using a thicker shell to cover NPs by traditional silicates, the chemocatalyst was able to work well, despite the presence of the enzyme in solution, while the inhibition of TADH was only 10%.

Comparing the results (Table 2) of all the methods tested for a possible combination of NPs and TADH in the double reduction of 3-methyl-2-cyclohexenone, leading to an optically pure saturated alcohol, the best result, i.e., 36% of (1S,3S)-3-methylcyclohexanol, was attained by performing the reaction with TADH and Pd NPs covered by a traditional silica shell. The incomplete conversion was likely due to the TADH inhibition (10%) from the interaction of the protein-silica layer, as discussed in the literature [49].

In the case of the reaction performed by free TADH and Pd NPs encapsulated with a thin DGS layer, the final yield was 12%, as in the case in which the two catalysts were both used without any protection, likely the thickness of the DGS-covered NPs was too small to be effective in blocking the interface with the NPs surfaces. In the case of the enzyme entrapped in the sol-gel, the NPs work well, but the reaction was slowed down, while the yield of the enzyme-catalyzed reaction is 24% less, since the protein was not free to have its natural conformation inside the sol-gel network. A less rigid sol-gel for the protein, i.e., with a larger mesh, definitely affects the NPs reactivity, as the NPs can permeate it, thus allowing an effective interaction with TADH. The same result (~19% of yield) was obtained by entrapping the NPs in the sol-gel, which, in allowing quantitative conversion, also worsened the inhibition due to the interactions of TADH with the large surface of sol-gel. The last attempt to insert a barrier between TADH and NPs was to use total cells of *E. coli* to entrap TADH, but the approach failed completely, because the cell membranes were not permeable to the reagents, or the heterogeneous solution did not allow good NPs activity.

## 3. Materials and Methods

### 3.1. Materials

The lignosulfonate sample, as ammonium derivative, was a courtesy of Burgo Group S.p.A. (Tolmezzo, Italy). NADH was obtained from Fluka, PdCl_2_ and H_2_PtCl_6_ x 6H_2_O from Strem while all the other chemicals were purchased from Sigma-Aldrich.

### 3.2. Instruments

Gas chromatography system used was a Shimadzu GC-2010 (Shimadzu, Kyoto, Japan), plus a system (Shimadzu, Kyoto, Japan) using He as carrier gas and an Agilent CP-Chirasil-DEX CB column (25 m × 0.32 mm × 0.25 μm) (Agilent, Santa Clara, CA, USA).

UV-Vis spectra were recorded by UV-3600 UV-VIS-NIR spectrophotometer (Shimadzu, Kyoto, Japan).

Circular dichroism spectra were measured using a cylindrical 1-mm Hellma Quartz Suprasil cuvette in a Jasco J-720 spectropolarimeter, in the wavelength range of 170 nm–270 nm at room temperature. The measuring conditions were: band width, 0.5 nm; response, 4 s; data pitch, 0.2 nm; scanning speed, 50 nm/min; and number of accumulations, 5. The baseline obtained with the phosphate buffer (0.05 M, pH 7.2) was subtracted from the sample spectra. Molar ellipticities (in deg cm^2^ dmol^−1^) were plotted against the wavelength.

DLS instrument was a Zetasizer Nano ZS (Malvern, Etten Leur, Netherlands).

XPS analysis was carried out using a Varian instrument with a double anode (Mg+Al) TA10 X-ray energy source (VSW Scientific Ltd., Manchester, UK) with a HA100 hemispheric analyzer (VSW Scientific Ltd., Manchester, UK) with a single channel recorder (Varian mod. 981–2043 ion cannon with variable energy of 0–3000 V).

Size-exclusion chromatography-HPLC analysis was carried out using HPLC with a Bio-Sil SEC 125 column (exclusion limit, ~80,000; column dimensions, 7.8 × 300 mm; molecular protein range, 5000–100,000; particle size, 5 μm; protein capacity, 0.01–1.5 mg; pH range, 2–8). Mobil phase was a buffer containing 0.05 M Na_2_HPO_4_, 0.05 M NaH_2_PO_4_, 0.15 M NaCl, pH 6.8. The flow rate was 1.0 mL/min, and the amount of sample injected was 20 μL. The detection UV was set at 280 nm, with the maximum wavelengths of absorption at 320 nm and emission at 523 nm.

### 3.3. Methods

Lignin-stabilized Pd and Pt NPs were prepared according to the procedure previously published [29]; briefly 0.06 g of lignin was placed in 10 mL of water, then 0.01 g of PdCl_2_ was added (or 0.06 g of lignin, 0.03 g of H_2_PtCl_6_·6H_2_O in 6 mL of water) and the solution was heated at 80 °C for 3 h in aerated conditions. Concentrations, referred to the metal content, are: 5.64 × 10^−3^ M for Pd and 9.66 × 10^−3^ M Pt NPs of larger diameters were obtained extending the reaction time from 3 h to overnight. The diameters values, obtained by DLS measures, were 8 nm (3 h) and 19 nm (overnight) for Pt and 8 nm (3 h) and 14 nm (overnight) for Pd. Formation of NPs was evidenced by the change of the color of the solution (from brown to black).

Synthesis of DGS-Pd NPs was carried out mixing together 0.1 g of previously synthesized DGS [46], 0.1 mL of Pd NPs solution, and 0.1 mL of deionized water.

Synthesis of silica shell Pd NPs was carried out by adjusting the pH of NPs solution (10 mL) to neutral with 0.1 M NaOH, and then adding 1.5 mL of TEOS and 0.1 mL of APTES under vigorous stirring for 10 min; the mixture was then left static at room temperature for 2 h.

Sol-gel was made using TEOS and TMOS or MTMS in mixture with APTES; alternatively, it was made employing DGS. Initially, 8.90 mL TEOS or TMOS and 2.2 mL APTES or MTMS were mixed with acidic water (1.4 mL, pH 2.8, adjusted with HCl) and 10.4 mL distilled water. The mixture was sonicated for 10 min and kept in a freezer overnight. The day after the produced ethanol (from the hydrolysis of silicates) was evaporated at reduced pressure, and then the solution was brought to the same initial volume by adding water. The condensation step started by adding to the previously prepared solution, Pd NPs solution in a ratio of 1:1 or 1:2 (sol: NPs solution) and making the mixture basic with aqueous NaOH.

Instead, for the TADH immobilization in sol-gel, the enzyme solution was added to sol in the ratio of 1:1, without any addition of base (in 0.05 M phosphate buffer solution, pH 7.2). DGS sol-gel was synthesized as follows: 110 mg of DGS and 150 μL of water were mixed by sonication at 0 °C for 10 min to obtain a homogenous solution, then 200 μL of buffer (phosphate, 0.05 M, pH 7.2) TADH solution or 200 μL of Pd NPs aqueous solution were added (the NPs aqueous solution was made by mixing 10 μL of initial NPs solution and 190 μL of water, then some drops of diluted HCl were added until the pH resulted neutral). The formation of sol-gel occurred after 5 min leaving the solution static.

Purification of TADH was performed according to the methodology developed by Höllrigl et al. [37] from cultivation of recombinant *E. coli* BL21 pLysS pASZ2 [51]. *E. coli* BL21 (DE3) harboring pASZ2 (pET 11a derivative that contains the gene for TADH) was cultivated using the Overnight Express^TM^ Autoinduction System (Novagene), for 24 h at 37 °C. The cell pellet was re-suspended in pH 7.2 buffer (0.05 M) and incubated for 20 min at 80 °C. The supernatant after ultracentrifugation contained more than 90% of pure TADH, as estimated from SDS-PAGE, and it was used as it (250 μg/mL).

The multistep reaction was performed carrying out first the reduction of 3-methyl-2-cyclohexenone to 3-methylcyclohexanone adding 10 μL Pd NPs or 5 μL Pt NPs solution and 2 mL of phosphate buffer solution (pH 7.2, 0.05 M) containing 3-methyl-2-cyclohexenone in a concentration of 50 mM; the solution was left in autoclave, under 10 bar of H_2_ at 30 °C for 4 h (Pt NPs) or for 8 h (Pd NPs). After the reduction time conducted in autoclave, to the depressurized previous reaction mixture solution, 200 μL of TADH solution (250 μg/mL), 1.4 mg of NADH, 4 mg of GDH and 36 mg of glucose were added in the same pot, and the resulted final mixture was kept for 20 h at 30 °C in open air to achieve the selective reduction of (S)-3-methylcyclohexanone to (S,S)-3-methylcyclohexanol. At the end of the reaction, the mixture was extracted with 1:5 (*v*/*v*) ethyl acetate, and the organic phase was dried over Na_2_SO_4_ before GC analysis.

The one-flask double reduction reaction was carried out in autoclave, under 10 bar of H_2_ for 24 h at 30 °C. The starting reaction mixture was prepared by adding 10 μL Pd NPs (or 5 μL Pt NPs) solution, 200 μL of TADH solution (250 μg/mL), 1.4 mg of NADH, 4 mg of GDH and 36 mg of glucose in 2 mL of phosphate buffer solution (0.05 M, pH 7.2) containing 3-methyl-2-ciclohexenone (50 mM). At the end of the reaction, the mixture was extracted with 1:5 (*v*/*v*) ethyl acetate, and the organic phase was dried over Na_2_SO_4_ before GC analysis.

In the case of GDH, we followed the increasing of the 340 nm absorption band of NADH, then evaluating the enzyme activities before and after incubations in presence of NPs (high and low conc., see Figure 1), glucose (0.1 mM) and NAD^+^ (0.1 mM), over 100 s at room temperature and under different NPs concentrations. The final volume was made to 4 mL adding a phosphate buffer at pH 7.2.

In the case of TADH (see Figure 2 and Figure 3), we followed the decreasing of the 340 nm absorption band of NADH (initial conc. 0.1 mM), then evaluating the enzyme activities before and after incubations in presence of NPs (10 μL of Pt NPs 9.66 × 10^−3^ M or 20 μL of Pd NPs 5.64 × 10^−3^ M), glucose (0.1 mM), and 3-methyl cyclohexanone (0.1 mM), over 100 s at room temperature. The final volume was made to 4 mL adding a phosphate buffer at pH 7.2.

DLS was used to monitor the hydrodynamic radius of NPs and NPs-TADH using the Pd diffractive index (1.7229). Samples for DLS analyses were prepared by diluting the NPs solutions (about 2000-fold) and filtering (PTFE, 0.2 μm) them. Experiments to observe changes in TADH dimensions were made using the common protein diffractive index (1.33) and following the values during the time (max 10 min).

Samples for XPS analyses were prepared by filtering the solution of TADH incubated with the NPs (at two ratios: 100 μL of TADH solution with 15 or 25 μL Pd NPs solution) using Amicon Ultra-2 mL 100 kDa centrifuge cut-off filters. The heavy fractions collected on the filters were then washed with deionized water and the residues were re-suspended in water; the solid powders, suitable for XPS analyses were finally obtained by an evaporation step conducted in a Speed Vac apparatus.

SEC analyses were conducted on solutions of TADH and Pd NPs after incubation time of 24 h (100 μL TADH solution, with 5, 15 and 25 μL Pd NPs solution). The activity test of the nanobioconjugate collected from this SEC analysis (peak from 7 to 9 min) was performed by adding cyclohexanone (10 mM) and NADH in stoichiometric amounts for 14 h at 30 °C.

Two colorimetric tests, namely ninhydrin and Bradford, were adopted to check the presence of proteins in the buffer solution. Ninhydrin test was used when in presence of the phosphate buffer (0.05 M, pH 7) since the Bradford test, in such experimental conditions, can produce false positives results.

## 4. Conclusions

It is not simple to join the world of macromolecules, such as proteins, and very different metal nanostructures. Both these amino acidic polymers, with their well-defined 3D structures, and the metal colloid solutions, have catalytic properties, but each has been used under different conditions, and for different reasons. The present experiments have clearly shown that integration between these two different approaches in catalysis was possible, even if significant problems and challenges must be overcome. In this article, several studies have been discussed, and some assumptions about the interactions between a chemocatalyst and a biocatalyst have been proposed. However, it was still difficult to determine the specific rules that might govern the adsorption and conformation of biomolecules on NPs [52].

The combination of TADH and Pt or Pd NPs was stabilized by the lignin, while the size of the NPs resulted, among other critical parameters, in mutual inhibition, where the bigger the NPs were, the more harmful they were for the enzyme, even if the NPs themselves were only moderately inactivated. Conversely, the smaller NPs resulted in a minimal TADH denaturation; however, they were dramatically inhibited. In conclusion, the chemocatalysts were found to be very sensitive to deactivation, which was not related to the amount of enzyme used, while the inhibition of the biocatalyst can be strongly reduced by minimizing the NPs/TADH ratio used to catalyze the reaction. Some methods to avoid direct binding of NPs with TADH were tested, and the best results were obtained by using large Pd NPs, as well as protecting their surfaces with a silica shell. This method was simple and versatile, and could be applied to many NPs; furthermore, the interest about this tandem reduction reaction in the presence of metal NPs and enzyme concerns not only the production of organic fine chemicals but also the new fields of biomass transformations for chemical and energy uses [53,54].

## Figures and Tables

**Table 1:** Effect of H_2_ pressure on conversion and selectivity of the Pd and Pt-catalyzed reduction of 3-methyl-2-cyclohexenone to 3-methylcyclohexanone. Reaction conditions: 3-methyl-2-cyclohexenone 50 mM in 2 mL phosphate buffer solution; 5 μL of 9.66 × 10^−3^ M Pt NPs solution or 10 μL of 5.64 × 10^−3^ M Pd NPs solution, 30 °C, 4 h for Pt NPs catalyzed reaction, 8 h for the Pd NPs catalyzed reaction.

NPs	P_H2_ (bar)	Conversion (%)	Selectivity (%)
Pd	5	50	49
	10	95	94
	20	95	94
Pt	5	99	90
	10	99	84
	20	80	66

**Table 2:** 3-(1S,3S)-Methylcyclohexanol (from GC data) yields for the different methods used to perform one-flask reduction of 3-methyl-2-cyclohexenone.

Conditions	Overall Yield (%)
Free TADH and free Pd NPs	14
TADH in traditional sol-gel and free Pd NPs	19
TADH in DGS sol-gel and free Pd NPs	23
Free TADH and Pd NPs in DGS sol-gel	19
Free TADH and DGS-coated Pd NPs	12
Free TADH and traditional silica-coated Pd NPs	36
